# Complete mitochondrial genome of a living wild-type Chinese giant salamander *Andrias davidianus* (Amphibia: Cryptobranchidae) in Huangshan

**DOI:** 10.1080/23802359.2016.1197078

**Published:** 2016-07-23

**Authors:** Jing-Cheng Xu, Dian-Cheng Yang, Yan-Rong Chen, Qi-Neng Wu, Song Huang

**Affiliations:** a College of Life and Environment Sciences, Huangshan University, Huangshan, P.R. China;; b Xiuning Institute of Rare Aquatic Animals, Huangshan, P.R. China

**Keywords:** *Andrias davidianus*, mitochondrial genome, phylogenetic

## Abstract

The Chinese giant salamander, *Andrias davidianus*, is the biggest extant amphibian in the world. The population from Huangshan is distinct from other populations. The complete mitochondrial genome of a living wild-type Chinese giant salamander from Huangshan was sequenced. The total length is 16,565 bp, containing 13 protein-coding genes, 22 tRNA genes, 2 rRNA genes and a D-loop. The phylogenetic tree of *A. davidianus* and 12 other closely species belonging to the order Caudata was reconstructed.

The family Cryptobranchidae has a combined Asian/North American distribution, but contains only three species (*Andrias davidianus* in China, *A. japonicus* in Japan, *Cryptobranchus alleganiensis* in North America). The Chinese giant salamander, *A. davidianus*, is the biggest extant amphibian in the world (Fei et al. 2006). A complete mitogenome of an individual (origin unknown) from Longsheng Chinese Giant Salamander Breeding Center in Guangxi province had been determined (Zhang et al. 2003).

The population from Huangshan is distinct from other populations, indicating the existence of localized divergence (Murphy et al. 2000). In the summer of 1994, a giant salamander was collected from Qimen County (29.75°N, 117.84°E), Anhui Province, China, and has been feeding in the Xiuning Institute of Rare Aquatic Animals (Voucher number: HS16091). During the time of experiment, its weight was 11.25 kg, total length was 1.20 m. In this article, the complete mitochondrial genome of this wild-type Chinese giant salamander has been determined and described in order to provide basic genetic information about this species.

The complete mitogenome of *A. davidianus* (Genbank accession number KX268733) was sequenced to be 16,565 bp, which consisted of 13 typical vertebrate protein-coding genes, 22 transfer RNA (tRNA) genes, 2 ribosomal RNA (rRNA) genes and 1 D-loop. The overall base composition of the entire genome was as follows: A (31.9%), T (32.7%), C (21.0%) and G (14.4%). All the 13 protein-coding genes initiated with ATG as the start codon, except for COI, which began with GTG. Nine protein-coding genes ended with complete stop codons (TAA, TAG, and AGG), and the other four genes terminated with T–– as an incomplete stop codon, which was presumably completed as TAA by post-transcriptional polyadenylation (Anderson et al. 1981). Among the mitochondrial protein-coding genes, the ATP8 was the shortest, while the ND5 was the longest. The 22 tRNA genes ranged in the size from 66 to 76 bp. The lengths of 12S and 16S rRNA are 925 bp and 1575 bp. The D-loop in size is 769 bp. As in most vertebrates, a non-coding spacer between the tRNA-Thr and tRNA-Pro genes is also found in *A. davidianus* (384 bp).

Mitochondrial genome sequences of *A*. *davidianus* in this study, and together with other 12 related species was used to perform the phylogenetic analysis. A maximum likelihood tree was constructed using online tool RAxML (Stamatakis et al. 2008) (Figure 1). The phylogenetic analysis result was consistent with the previous research (Matsui et al. 2008).

**Figure 1.**
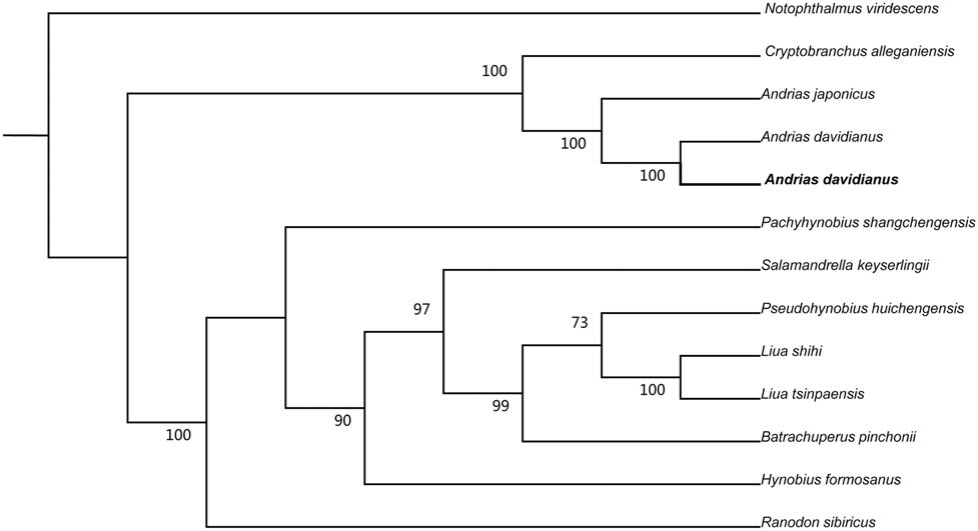
A maximum likelihood (ML) tree of the 13 species from Caudata was constructed based on the dataset of mitochondrial genome by online tool RAxML. The numbers above the branch meant bootstrap value. Bold black branches highlighted the study species and corresponding phylogenetic classification. The analyzed species and corresponding NCBI accession number are as follows: *Notophthalmus viridescens* (EU880323), *Cryptobranchus alleganiensis* (GQ368662), *Andrias japonicus* (AB208679), *Andrias davidianus* (AJ492192), *Andrias davidianus* (KX268733), *Pachyhynobius shangchengensis* (DQ333812), *Salamandrella keyserlingii* (JX508762), *Pseudohynobius huichengensis* (FJ532060), *Liua shihi* (DQ333810)*, Liua tsinpaensis* (KP233806), *Batrachuperus pinchonii* (KP122337), *Hynobius formosanus* (DQ333816), *Ranodon sibiricus* (AJ419960).
